# Machine Learning Prediction of Excess Relative Risk for Radiation-Induced Solid Thyroid Cancer Among Nuclear Medicine Healthcare Professionals: A Computational Modeling Study

**DOI:** 10.3390/bioengineering13060696

**Published:** 2026-06-18

**Authors:** Mariem Chouchen, Chamseddine Barki, Ismail Dergaa, Halil İbrahim Ceylan, Andrea de Giorgio, Nicola Luigi Bragazzi, Hanene Boussi Rahmouni

**Affiliations:** 1 Research Laboratory of Biophysics and Medical Technologies, The Higher Institute of Medical Technologies, University of Tunis El Manar, Tunis 1002, Tunisia; chouchenemariem02@gmail.com (M.C.); chamseddine.barki@istmt.utm.tn (C.B.); hanene.boussi@istmt.utm.tn (H.B.R.); 2 High Institute of Sport and Physical Education of Ksar Said, University of Manouba, Mannouba 2010, Tunisia; phd.dergaa@gmail.com; 3 Physical Activity Research Unit, Sport and Health (UR18JS01), National Observatory of Sports, Tunis 1003, Tunisia; 4 High Institute of Sport and Physical Education of Kef, University of Jendouba, Jendouba 7100, Tunisia; 5 Physical Education and Sports Teaching Department, Faculty of Sports Sciences, Atatürk University, Erzurum 25240, Türkiye; 6 Artificial Engineering, 80121 Naples, Italy; andrea@degiorgio.info; 7 Department of Clinical Pharmacy, Saarland University, 66123 Saarbrücken, Germany

**Keywords:** absorbed dose, computational modeling, decision tree, excess relative risk, I-131 exposure, machine learning, multilayer perceptron, nuclear medicine, occupational exposure, random forest, thyroid cancer

## Abstract

**Background**: Nuclear medicine healthcare professionals (NMHP) sustain chronic occupational exposure to iodine-131 (I-131), conferring an elevated risk of radiation-induced solid thyroid cancer. Established radiobiological prediction tools derive risk coefficients from atomic bomb survivor data but are not configured for rapid individualized risk assessment in occupational exposure settings. This study examined whether machine learning algorithms can serve as high-precision computational surrogates for excess relative risk estimation in NMHP. **Aim**: The study aimed to (i) develop and validate three machine learning algorithms for predicting the excess relative risk per unit absorbed dose for radiation-induced solid thyroid cancer (ERR/Gy.RST), (ii) characterize relationships between dosimetric and demographic features and predicted risk, and (iii) identify the optimal algorithm for deployment in occupational health surveillance. **Methods**: A dataset of 4657 observations was constructed from Life Span Study-derived ERR/Gy parameters, adapted to occupational low-dose conditions, using a dose-and-dose-rate effectiveness factor of 2.0, per ICRP Publication 103. Five features (gender, age at exposure, current age, distance from the I-131 source, and cumulative absorbed dose in the thyroid) were used to train a decision tree regressor (dtcr), a random forest regressor (rfr), and a multilayer perceptron (MLP) neural network algorithm. **Results**: Cumulative absorbed dose in the thyroid correlated positively with ERR/Gy.RST (r = 0.63, *p* < 0.01), while radiation source distance demonstrated a strong inverse association (r = −0.38, *p* < 0.01). The MLP algorithm achieved R^2^ score = 0.999, MSE = 0.002, and MAE = 0.010, substantially outperforming the rfr (R^2^ score = 0.700, MSE = 0.410, MAE = 0.295) and the dtcr (R^2^ score = 0.510, MSE = 0.654, MAE = 0.289). **Conclusions**: The MLP algorithm provides a high-fidelity surrogate for established ERR/Gy.RST projection tools in the NMHP context, enabling computationally efficient, feature-integrated occupational radiation-induced thyroid cancer risk quantification. These findings suggest that machine learning-based surrogate modeling is a practical, scalable complement for occupational health practitioners and radiation protection officers to support individualized surveillance of radiation-induced thyroid cancer risk in nuclear medicine departments.

## 1. Introduction

Thyroid cancer is the most prevalent endocrine malignancy globally, with an estimated 44,020 new cases diagnosed in the United States in 2025 alone [1]. Its incidence has risen consistently over three decades, a trend driven partly by increased diagnostic sensitivity, and partly by genuine epidemiological shifts linked to environmental and occupational exposures [2,3,4]. Among identified carcinogens, ionizing radiation is particularly relevant as a thyroid-specific risk factor, a relationship established with exceptional consistency across epidemiological studies spanning six decades [5,6]. The thyroid gland ranks among the most radiosensitive tissues in the human body, especially at younger ages, a characteristic exploited therapeutically in the treatment of thyroid cancer and hyperthyroidism with iodine-131 (I-131), but which simultaneously creates carcinogenic risk in occupationally exposed populations [7,8]. Evidence from the Life Span Study (LSS) of atomic bomb survivors conducted by the Radiation Effects Research Foundation (RERF), the Chornobyl accident follow-up studies, and multiple medically irradiated cohorts has consistently demonstrated dose-dependent increases in thyroid cancer incidence following external gamma or internal radioiodine exposure [9,10,11,12]. These findings have shaped international radiation protection frameworks, including the 2007 recommendations of the International Commission on Radiological Protection (ICRP), which define occupational dose limits and tissue-specific risk coefficients for solid cancer projection [13,14]. The magnitude of radiation-induced thyroid cancer risk varies substantially by current age, age at exposure, gender, distance from radiation ionization source, and cumulative absorbed dose, and childhood or adolescent exposure confers the highest susceptibility [15]. Translating these relationships from the acute, high-dose atomic bomb survivor context to the chronic, low-dose occupational setting demands both radiobiological precision and practical deployment infrastructure.

Within nuclear medicine (NM), NM healthcare professionals (NMHP) represent a population of particular occupational exposure concern. Metabolic therapy with I-131 constitutes the therapeutic cornerstone for differentiated thyroid cancer and a first-line treatment for hyperthyroidism, generating substantial radioiodine activity in NM units globally [16,17,18]. During the preparation, administration, and post-treatment monitoring phases of I-131 therapy, NMHP are exposed to external gamma radiation emitted by I-131 (principal photon energy 364 keV) and to the risk of internal contamination from inhalation of volatile radioiodine [19]. Bioassay studies in NM departments have documented measurable thyroid I-131 burdens among NMHP following routine handling of radioiodine, confirming the reality of internal contamination pathways even under standard radiation protection protocols [19]. The excess relative risk (ERR) per 100 mGy for thyroid cancer among NMHP has been estimated at −0.06 (95% CI: −0.08 to 0.39), a figure reflecting genuine statistical uncertainty inherent in low-dose dose–response estimation [20,21]. Furthermore, an increased relative risk of solid cancer has been observed in relation to the age, the age at exposure, and the gender of the NMHP [20,21,22,23]. The LSS provides the most comprehensive empirical basis for radiation-induced carcinogenesis risk coefficients among NMHP [9,10]. The RadRAT radiation risk assessment tool and the LARisk (R package) operationalize LSS-derived ERR coefficients by applying dose- and dose-rate-effectiveness-factor (DDREF) corrections to project lifetime cancer risk under chronic low-dose-rate occupational exposure conditions [24,25]. These tools and frameworks demonstrate value for NMHP-level risk projection; their practical application in individualized, real-time occupational surveillance, however, remains constrained by computational architecture and the specialized radiological expertise required to operate them.

The integration of machine learning into radiation protection science represents a rapidly expanding frontier [26,27]. Several specific gaps impede its progress in the NMHP context. Most predictive models for radiation-induced cancer risk have been calibrated on high-dose, acute-exposure cohort data without systematic adaptation to the chronic, fractionated, low-dose-rate profile characteristic of NMHP [28,29]. The dose–response relationship at occupationally relevant doses below 100 mGy remains scientifically contested, with ongoing debate between the linear no-threshold model and threshold or hormetic alternatives, a controversy that conventional single-model risk tools cannot accommodate [30,31]. Published machine learning applications in radiation-induced cancer have predominantly addressed treatment planning and normal tissue toxicity prediction rather than the occupational-exposure-induced carcinogenesis [32,33,34]. The multivariate interaction between individual demographic variables, gender, current age, age at exposure, occupational dosimetric parameters (such as cumulative absorbed dose and dose rate), and source proximity has not been systematically modeled in a form deployable within the existing occupational exposure assessment framework in NM [35,36]. No validated, computationally efficient tool currently exists for rapid, feature-integrated ERR estimation specifically calibrated to I-131 handling environments. A surrogate machine learning model that accurately reproduces established ERR projections while operating at computational speeds compatible with routine occupational health monitoring would address this gap concretely.

To address these limitations, the present study developed and validated a machine learning model to predict the ERR per unit of absorbed dose for radiation-induced solid thyroid cancer (ERR/Gy.RST) among NMHP handling I-131 in metabolic therapy. The novelty of this work lies in integrating epidemiological risk models derived from the Life Span Study (LSS) with a machine learning approach adapted to occupational low-dose exposure conditions, using the DDREF correction (ICRP Publication 103) [13]. Unlike conventional radiation-induced thyroid cancer risk assessment tools that rely on static epidemiological equations or cohort-based estimations, the proposed framework combines multivariate machine learning modeling with individualized occupational exposure parameters to enable dynamic and feature-integrated ERR/Gy.RST prediction. This enables a more precise and context-specific estimation of radiation-induced thyroid cancer risk in NM settings.

In the present study, three machine learning algorithms were developed and benchmarked. The objectives of this study were to: (i) develop and validate each algorithm’s predictive performance for ERR/Gy.RST, (ii) quantify the relationships between dosimetric and demographic features and the risk of developing a radiation-induced solid thyroid cancer, and (iii) identify the optimal algorithm for deployment as an intelligent radiation-induced thyroid risk assessment tool in NM. By transforming established radiobiological risk equations into an intelligent predictive framework compatible with routine occupational monitoring, this study provides a methodological bridge between classical radiation epidemiology and artificial intelligence (AI)-driven radiation protection systems.

## 2. Materials and Methods

In the present study, three machine learning algorithms were developed and benchmarked. The methodological contribution of this work consists of: (i) constructing a computational surrogate model derived from LSS-based ERR/Gy.RST formulations corrected with DDREF factors, (ii) integrating demographic and dosimetric variables into a unified predictive framework, and (iii) evaluating the comparative performance, robustness, and deployment suitability of multiple machine learning algorithms for occupational radiation-induced thyroid cancer risk assessment.

### 2.1. Data Source and Dataset Generation

This study used publicly released RERF datasets consisting of publicly available, de-identified data maintained by the RERF under established data access agreements with the Japanese Ministry of Health, Labor, and Welfare (MHLW) and the U.S. Department of Energy (US DoE). The study exclusively used de-identified aggregate epidemiological data. As no direct human participants were involved and all data originated from aggregated, anonymized secondary sources held by a public research institution, formal institutional ethics committee review was not required. The required acknowledgment statement was included in accordance with RERF guidelines.

In this study, the dataset was used as a primary open-access source to estimate thyroid cancer risk and to generate a derived dataset for model development. ERR/Gy.RST values for our dataset observations were computed using thyroid cancer ERR/Gy model coefficients as implemented in the RadRAT radiation risk assessment tool [24]. Complete specifications of the ERR model structure, including the baseline ERR/Gy coefficient, age-at-exposure modifiers, attained-age modifiers, and gender-specific parameters, are documented in the primary RadRAT publication [24]. In contrast to the original RadRAT implementation, which is primarily designed for conventional epidemiological risk projection, the present study transformed these radiobiological equations into a machine learning-compatible computational dataset suitable for rapid individualized occupational risk prediction in I-131 units.

Although the present analysis relies primarily on radiation risk models derived from the LSS cohort of Hiroshima and Nagasaki atomic bomb survivors, this choice was motivated by the fact that the LSS remains the most comprehensive and internationally recognized epidemiological resource for quantifying radiation-induced cancer risks [13,14,24]. The LSS dataset provides long-term follow-up data with detailed stratification by absorbed dose, sex, age at exposure, and attained age, and constitutes the scientific basis for major international radiation protection frameworks, including those of the ICRP, International Atomic Energy Agency (IAEA), the United Nations Scientific Committee on the Effects of Atomic Radiation (UNSCEAR), and the Biological Effects of Ionizing Radiation (BEIR) [13,14,24]. U.S.-associated contributions were incorporated through the RadRAT implementation and the broader radiological risk assessment framework developed under the U.S [24]. National Cancer Institute and the U.S. Department of Energy collaborations with RERF. The combined Japanese and U.S.-associated resources were therefore selected because they represent the current international reference standard for radiation-related cancer risk estimation.

To generate an NMHP-representative dataset, acute LSS-derived ERR/Gy coefficients from the Japanese atomic bomb survivor cohort (the survivors of Hiroshima and Nagasaki) were used as primary data sources for radiation-induced cancer risk models [13]. These coefficients were converted to the chronic low-dose-rate occupational setting by applying a DDREF of 2.0, consistent with ICRP Publication 103 recommendations for radiation-induced solid cancer risk at occupationally relevant dose rates [13]. This methodological adaptation represents a key contribution of the present work. This adjustment halves the ERR/Gy coefficient to reflect the reduced biological effectiveness of chronic, fractionated exposures compared with acute, high-dose irradiation.

In addition, the population transferability of LSS-derived ERR models to NMHP may be influenced by demographic, genetic, and healthcare-related differences between Japanese survivors and occupationally exposed healthcare workers in other regions. The present study focuses exclusively on external gamma radiation exposure and thyroid cancer risk estimation; therefore, the findings should not be generalized to other radiation types, mixed exposure scenarios, or other cancer endpoints.

Five input features were defined to represent the occupational exposure profile of NMHP working in the I-131 units: gender, distance from the I-131 gamma radiation source, age at exposure, current age, and cumulative thyroid gland absorbed dose. For each combination of the five attributes, we calculated and predicted the corresponding ERR/Gy.RST. Exhaustive combinations of these features across their NMHP-relevant ranges generated a final dataset of 4657 observations. Python programming language (version 3.10) was used for all data processing, model fitting, and analysis. Table 1 provides an overview of the variables used in this study.

### 2.2. Data Preparation

In our study, we generated a new dataset with 5 input features and 1 output feature for ERR/Gy.RST prediction, as illustrated in Table 1. Furthermore, we restricted our population analysis to an age-specific range of exposure for NMHP. In this study, we focused exclusively on gamma radiation dose, as NMHP are exposed to gamma radiation during I-131 therapy. The cumulative absorbed dose to the thyroid was assumed to result only from low-dose-rate exposure. This assumption is justified by the nature of occupational exposure in NM, where radiation is continuously received at low dose rates over extended periods, rather than in acute high-dose exposures. Additionally, we added columns estimating the ERR/Gy.RST. To estimate ERR/Gy.RST, we utilized the prediction formulas developed in the study by Berrington de Gonzalez et al. [24]. The resulting dataset therefore represents a hybrid computational framework combining established epidemiological ERR/Gy.RST equations with structured machine learning-ready feature engineering.

No missing values are present in the dataset, as all values were derived from deterministic formula outputs. Given the nature of the dataset, no feature scaling or transformation was necessary. Although scaling was not strictly required due to deterministic data, StandardScaler was applied to stabilize training. Multicollinearity was addressed by selecting the five input features to represent distinct, non-redundant aspects of occupational exposure.

### 2.3. Machine Learning Algorithm Selection

In our study, three algorithms were selected based on their complementary structural properties and documented performance in predicting radiation-induced cancer risk using tabulated datasets [32,33,34]: a decision tree regressor (dtcr) algorithm, providing a transparent, interpretable baseline through explicit rule-based partitioning of the feature space; a random forest regressor (rfr) algorithm, combining 300 decision trees through bootstrap aggregation to reduce prediction variance while preserving interpretable feature importance metrics [37]; and a multilayer perceptron (MLP) neural network algorithm, leveraging multiple hidden layers to model complex nonlinear feature interactions through universal approximation [38,39]. This architecture was specifically intended to approximate the linear relationships embedded in LSS-derived ERR/Gy.RST formulations under chronic low-dose-rate exposure conditions. Prior to training, all features were standardized using a StandardScaler, mapping each variable to a zero mean and unit variance to eliminate scale disparities and stabilize gradient-based optimization [40,41]. The architecture of our MLP algorithm was selected following several empirical tests. The goal of these tests is to balance the algorithm’s generalization potential with complexity. We experimented with a range of configurations in these studies, from shallow to deep (6 layers). Ultimately, we chose the final design since it performed better on the validation set in terms of precision and loss stability. Finally, our dataset was split into a 70% training set and a 30% test and validation set.

The architectures of the machine learning algorithms used in this study are presented in Table 2.

### 2.4. Hyperparameter Optimization

To optimize the hyperparameters of our algorithms, we employed the “GridSearchCV” with 4-fold cross-validation. For the dtcr algorithm, we set the maximum tree depth to 10, 20, 30, or 40; the minimum number of samples required for a split, and the pruning factor (ccp_alpha) to 0.5 or 0.7. For the rfr algorithms, we set the maximum tree depth to 100–200, the number of estimators to 300, and enabled the out-of-bag method to evaluate the algorithm’s internal performance and for accurate modeling [42,43]. The coefficient of determination (R^2^ score) served as the optimization metric for both tree-based algorithms. For the MLP algorithm, the Adam optimizer was employed with a learning rate of 0.1 and a batch size of 32. Although this learning rate exceeds the conventional default of 0.001 for the Adam optimizer (to prevent overfitting and underfitting), empirical testing on the present dataset confirmed stable loss convergence without oscillation, attributed to the smooth, deterministic nature of the ERR/Gy.RST function being approximated [44]. The loss function minimized during training was the mean squared error. Training proceeded for 300 epochs, with validation loss monitored at each epoch.

### 2.5. Performance Evaluation

Three metrics were evaluated and compared to algorithm performance on the held-out test set. The R^2^ score quantifies the proportion of the variance explained by the algorithm [45]. The mean absolute error (MAE) estimates the average absolute deviation between predicted and actual values [46]. The mean squared error (MSE) computes the mean of squared deviations, applying a greater penalty to larger prediction errors [47]. Predicted-versus-actual scatterplots and training/validation loss curves were generated for visual assessment of algorithm fit and learning stability.

**Figure 1:**
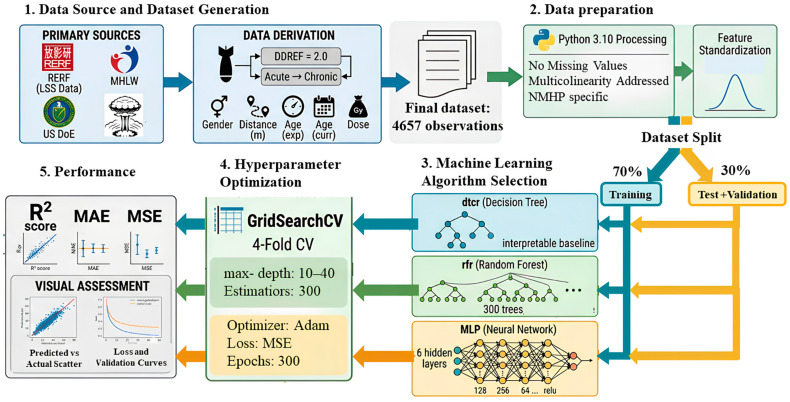
Machine learning pipeline for ERR/Gy.RST prediction.

To provide a clearer overview of our methodology, we present a schematic diagram of the machine learning pipeline used in this study (Figure 1). This figure illustrates the full workflow from data preparation, feature selection, and model training to hyperparameter optimization and performance evaluation.

### 2.6. AI Usage Statement

In preparing this manuscript, the authors used Claude (Anthropic, San Francisco, CA, USA) in February 2026 to improve the clarity and grammatical correctness of selected passages. The tool was used to revise the text for an academic tone, check for grammatical errors, and improve the quality of the English language. The authors did not use AI for data analysis, interpretation, or generation of scientific content. After using this tool, the authors thoroughly reviewed and edited all content and took full responsibility for the accuracy, integrity, and scientific validity of the work [48,49].

## 3. Results

### 3.1. Correlation Analysis

**Figure 2:**
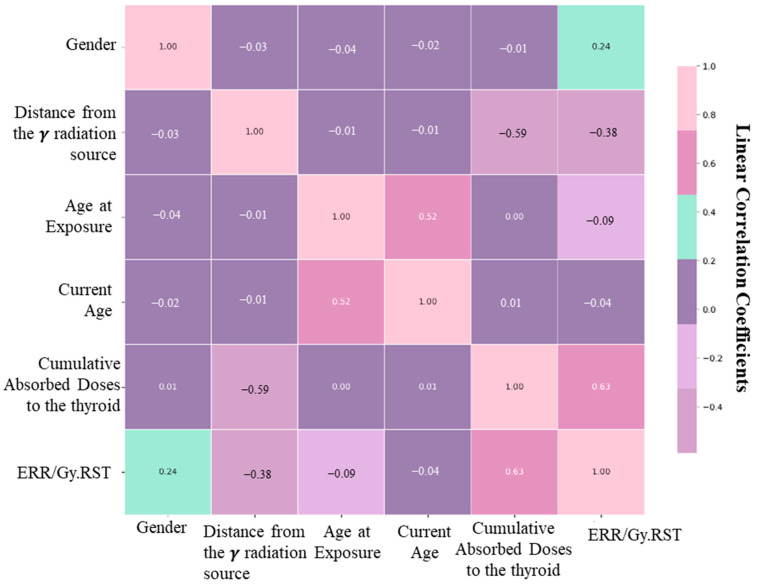
Correlation matrix for ERR/Gy.RST prediction.

Pearson correlation analysis across the 4657-observation dataset revealed a positive linear relationship between cumulative thyroid absorbed dose and ERR/Gy.RST (r = 0.63, *p* < 0.01). A strong inverse relationship was identified between distance from the gamma radiation source (emitted by the I-131 source) and ERR/Gy.RST (r = −0.38, *p* < 0.01). The positive correlation between NMHP gender and ERR/Gy.RST is consistent with the documented differential radiosensitivity of the thyroid gland by gender in the radiation-induced carcinogenesis literature. Correlation coefficients between age at exposure and current age of the NMHP and ERR/Gy.RST individually approached zero, indicating no linear marginal association when examined in isolation; however, nonlinear interactions may still be present. The complete pairwise correlation matrix for all variables is presented in the heatmap shown in Figure 2, which illustrates the full structure of linear relationships among all input features and the predicted outcome.

### 3.2. Algorithm Performance Comparison

Performance metrics for all three algorithms on the held-out test set are presented in Table 3, which summarizes R^2^ score, MSE, and MAE for each algorithm. The MLP algorithm achieved the highest predictive accuracy across all three metrics: R^2^ score = 0.999, MSE = 0.002, and MAE = 0.010. The rfr algorithm yielded R^2^ score = 0.700, MSE = 0.410, and MAE = 0.295. The dtcr algorithm achieved the lowest predictive performance with R^2^ score = 0.510, MSE = 0.654, and MAE = 0.289.

**Figure 3:**
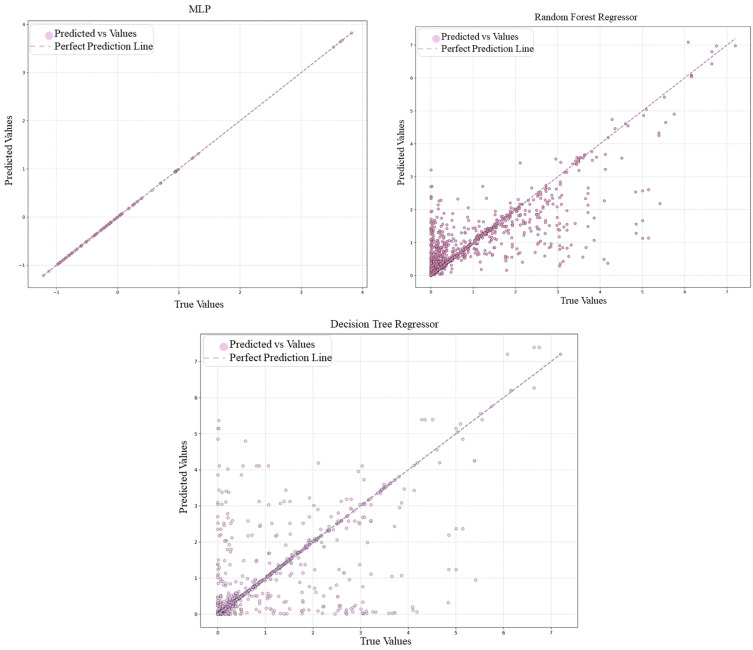
Correlation between the true and predicted ERR/Gy.RST values.

The scatterplots of predicted versus actual ERR/Gy.RST values for all three algorithms, presented in Figure 3, corroborate these numerical findings. The MLP data points are tightly distributed along the identity diagonal, indicating near-perfect agreement between predicted and actual values across the full test set range. Deviations from the identity line in the dtcr and rfr outputs correspond directly to their higher MSE and MAE values in Table 3, with the dtcr showing greater dispersion, particularly in the mid-range ERR/Gy.RST values.

### 3.3. MLP Learning Dynamics

**Figure 4:**
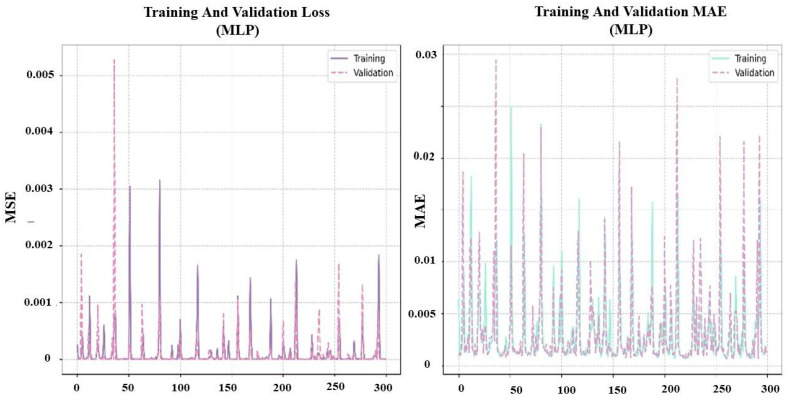
Loss and validation curves of the MLP algorithm for ERR/Gy.RST prediction.

Training and validation loss curves for the MLP algorithm over 300 training epochs are shown in Figure 4, illustrating convergence and generalization stability throughout the learning process. The training loss decreased monotonically and stabilized within approximately 50 epochs. The validation loss closely tracked the training loss throughout training, with no evidence of divergence at any epoch. The test loss and the training loss were comparable. This pattern of convergent training and validation losses confirms the absence of overfitting or underfitting phenomena, reflecting the combined effectiveness of the dropout regularization strategy (rate = 0.1), the StandardScaler normalization, and the 70/15/15 data partition.

## 4. Discussion

This study demonstrates that a six-layer MLP neural network trained on an NMHP-adapted LSS dataset achieves near-perfect surrogate modeling of ERR/Gy.RST (R^2^ score = 0.999, MSE = 0.002, MAE = 0.010), substantially outperforming both tree-based comparators. Distance from the I-131 source emerged as the strongest individual feature-level predictor (r = −0.38), with cumulative thyroid absorbed dose (r = 0.63) and gender providing additional predictive information. These results establish the MLP algorithm as a high-fidelity, computationally efficient surrogate for established radiobiological ERR/Gy tools in I-131 units in NM, and validate the utility of the NMHP-adapted LSS dataset as a training resource for this application.

### 4.1. Dose–Response Feature Relationships

The positive correlation between cumulative absorbed dose in the thyroid and the ERR/Gy.RST (r = 0.63, *p* < 0.01) aligns with fundamental radiobiological evidence that cumulative absorbed dose is the primary determinant of radiation-induced carcinogenesis, consistently demonstrated across the LSS and independent epidemiological cohorts [9,10,22]. The moderate magnitude of this correlation reflects the genuinely multivariate nature of thyroid cancer risk, shaped simultaneously by age at exposure, gender, and the biological effectiveness of exposure conditions. The even stronger inverse correlation between source distance and ERR/Gy.RST (r = −0.38, *p* < 0.01) reflects photon fluence attenuation governed by the inverse-square law, whereby doubling the separation distance from an unshielded point source reduces the dose rate fourfold [50]. Prior dosimetric surveillance studies in NM departments confirm that spatial distancing from I-131 preparation and administration areas is one of the most effective and immediately actionable dose-reduction strategies available to NMHP [51,52]. The identified gender correlation is consistent with the approximately two-to-three-fold higher radiation-related thyroid cancer incidence rate ratio in females compared to males, observed across the LSS, Chornobyl-exposed populations, and medically irradiated cohorts [7,8,15]. The near-zero marginal linear correlations for age at exposure and current age most likely reflect the genuine nonlinear, multiplicative nature of these age effects within ERR/Gy models, both variables interacting with dose in ways that linear correlation cannot capture, a complexity the MLP’s multilayer architecture resolves but that univariate analysis cannot. These findings indicate that future model iterations should include partial dependence analyses and interaction profiling to fully characterize age–dose relationships across the NMHP occupational exposure spectrum. While previous epidemiological investigations, including the LSS, the INWORKS cohort, and studies of medically exposed populations, have primarily focused on estimating population-level ERR/Gy.RST [7,8,9,10,22], relatively few studies have explored machine learning approaches for individualized radiation-induced thyroid cancer risk modeling in NMHP exposed to I-131. Most prior studies in NM and occupational protection have concentrated on dosimetric assessment, contamination monitoring, and radiation protection optimization rather than the predictive computational modeling of individualized ERR/Gy profiles [13,14,51,52]. Therefore, the present work extends the existing literature by bridging established radiobiological risk models with modern surrogate deep learning methodologies specifically adapted to occupational nuclear medicine settings.

### 4.2. Comparative Algorithm Performance

The MLP’s R^2^ score of 0.999, MSE of 0.002, and MAE of 0.010 on the held-out test set demonstrate near-complete surrogate-model fidelity. This level of performance is expected when a sufficiently expressive architecture learns data derived from a deterministic mathematical function; in this case, the RadRAT and LARisk ERR models. The scientific contribution lies not in the R^2^ score figure itself, but in three specific demonstrations: that the MLP captures the complete nonlinear input–output mapping of the ERR/Gy.RST function across the five-dimensional feature space; that the dtcr and rfr do not achieve this capture (R^2^ score = 0.510 and 0.700, respectively); and that the resulting surrogate is deployable as a standalone tool without requiring access to the full RadRAT or LARisk infrastructure. For comparison, biologically based deep learning models applied to the full LSS dataset for papillary thyroid cancer risk have reported R^2^ scores in the 0.90–0.93 range [53], with the gap to the present MLP algorithm reflecting both the deterministic data generation process employed here and differences in biological task complexity. The dtcr’s R^2^ score of 0.510 and the rfr’s R^2^ score of 0.700 reveal inherent limitations of tree-based architectures when confronted with smooth, continuous dose–response surfaces. Decision trees partition the feature space into axis-aligned hyperrectangles, introducing stepwise approximation errors wherever the true ERR/Gy.RST surface changes continuously with dose or distance [42]. The random forest reduces variance through ensemble averaging of 300 trees, but cannot overcome the fundamental non-smoothness of tree-based boundaries when approximating a differentiable curved surface in five dimensions [37]. The MLP’s ReLU-activated hidden layers enable piecewise-linear approximations of arbitrary precision, providing a structural advantage precisely for this type of smooth nonlinear regression task [38,39]. The convergence of training and validation loss curves throughout 300 epochs confirmed that the dropout regularization scheme (rate = 0.1) and the 70/15/15 partition produced a well-calibrated model without overfitting [41]. This result is supported by the loss trajectories in Figure 4, where training and validation loss remain closely aligned from early in the learning process through to full convergence.

Our approach aligns with prior deep learning applications using LSS-derived datasets for radiation-induced thyroid cancer risk estimation, where reported predictive performances generally ranged between R^2^ = 0.90 and 0.93 [54,55,56]. However, important methodological distinctions differentiate the present study from earlier work. Previous studies primarily focused on population-level cancer risk prediction or biological outcome classification using large epidemiological cohorts, whereas our study specifically targets occupational radiation exposure scenarios in NMHP handling I-131. In addition, earlier models rarely incorporated operational workplace exposure parameters such as source-to-worker distance, which emerged here as one of the strongest predictors of ERR/Gy.RST. By integrating occupationally relevant variables, including cumulative thyroid absorbed dose, age at exposure, current age, gender and working distance from radioactive sources, the present MLP algorithm provides a specialized framework tailored to NM radiation protection practice rather than general population radiation epidemiology. Furthermore, unlike many prior machine learning studies that focused on the direct prediction of observed cancer incidence, our study intentionally developed a surrogate model reproducing established radiobiological ERR/Gy formulations derived from RadRAT and LARisk. This distinction is important because it enables the rapid deployment of computationally efficient risk estimation tools while maintaining consistency with internationally recognized radiation risk assessment frameworks. In this respect, the present work complements the existing epidemiological and radiobiological literature by demonstrating that deep neural networks can accurately emulate complex ERR/Gy.RST equations for occupational decision support applications in nuclear medicine.

### 4.3. Occupational Health Implications

The MLP algorithm surrogate developed in this study provides occupational health practitioners with a rapid, deployable tool for ERR/Gy.RST estimation in NMHP, which requires only five parameters routinely captured in NM occupational health records: gender, current age, age at first occupational exposure to I-131, cumulative thyroid absorbed dose from dosimetric monitoring, and typical working distance from I-131 sources. Integrating this model into existing dosimetry monitoring systems would enable individualized radiation-induced thyroid cancer risk profiling during periodic occupational health reviews, an advancement over the current practice of applying population-average dose limits without personalized quantification of radiation-induced cancer risk [13,14]. The strong inverse correlation between distance and risk reinforces the systematic application of the ALARA principle through engineering controls [13]. Compared with conventional occupational radiation protection approaches, which generally rely on annual dose thresholds and retrospective dosimetric reporting, the proposed framework introduces a more individualized and predictive perspective consistent with recent trends toward precision occupational health and AI-assisted clinical decision support systems [57,58].

Remote-handling equipment for I-131 dispensing, automated dose calibrators, lead-glass shielding at preparation stations, and defined exclusion perimeters around active I-131 patients are supported as high-priority protective measures by the present data. The gender-related risk differential underscores the need for gender-stratified risk communication in NM occupational health programs, given the well-documented higher thyroid cancer incidence rate in radiation-exposed females [7,15]. These findings indicate that occupational health programs in NM departments should move toward individualized, machine learning-assisted risk stratification as a complement to population-level dosimetric monitoring, with targeted protective interventions prioritized for high-risk subgroups, including young female workers with proximity-based exposures and extended employment durations in high-activity I-131 units [57,58].

### 4.4. Limitations and Future Directions

Several methodological constraints warrant acknowledgment. The dataset used for model training is computationally generated from RadRAT and LARisk ERR/Gy.RST formula outputs rather than obtained from longitudinal follow-up of an NMHP cohort; the near-perfect MLP algorithm performance therefore reflects surrogate accuracy for a deterministic function, not the prediction of biological outcomes with inherent stochastic variability. Validation against prospective occupational epidemiological data, such as the INWORKS cohort [58] or the US Radiologic Technologists Study [20,21], is an essential next step before clinical and regulatory deployment. The model addresses the external gamma component of I-131 exposure represented through source-to-worker distance, while the internal thyroid dose from inhaled or ingested volatile radioiodine, a distinct and potentially dominant pathway for NMHP in high-volume I-131 units, is incorporated only through the aggregate cumulative absorbed dose in the thyroid feature. Future iterations should disaggregate external and internal dose contributions using bioassay-derived internal dose estimates as separate model inputs. The DDREF of 2.0, applied to translate acute LSS risk coefficients to the occupational low-dose-rate setting, introduces approximation uncertainty spanning roughly a factor of 2 to 10 across published analyses [28,29], and this uncertainty propagates into all generated ERR/Gy.RST values in the dataset. The model does not incorporate individual genomic, epigenetic, or immunological susceptibility factors, which emerging evidence identifies as significant modifiers of radiation-induced thyroid cancer risk [7]. The MLP’s multilayer architecture limits interpretability in regulatory contexts; future work should apply Shapley Additive exPlanations (SHAP) analysis [59] to generate quantitative, auditable feature attribution profiles that would substantially enhance regulatory acceptance. The dataset was constructed for a generic NMHP profile without stratification by specific occupational role, and role-stratified model development represents a high-priority extension of this work.

It should also be emphasized that our MLP algorithm is intended as a complementary tool to be used alongside existing methods for assessing the risk of radiation-induced solid thyroid cancer. It is not meant to replace traditional approaches, but rather to enhance and support them. Consequently, our assessment of radiation-induced cancer risk is limited to cross-sectional data. Moreover, our prediction model for radiation-induced solid thyroid cancer has not yet received authorization from health authorities and is not currently implemented in routine practice within radiation protection or NM departments.

## 5. Conclusions

This study examined the predictive performance of three machine learning algorithms, a dtcr, an RFR, and an MLP neural network, for estimating ERR/Gy.RST in NMHP, using a computational modeling approach grounded in LSS-derived ERR/Gy parameters adapted to occupational low-dose conditions via a DDREF of 2.0 per ICRP Publication 103. Across all performance metrics on the held-out test set, the MLP substantially outperformed both tree-based comparators, achieving R^2^ score = 0.999, MSE = 0.002, and MAE = 0.010, compared to maximum R^2^ values of 0.700 and 0.510 for the rfr and dtcr, respectively. Correlation analysis established distance from the I-131 source as the single strongest feature-level predictor of ERR/Gy.RST (R = −0.38, *p* < 0.01), with cumulative thyroid absorbed dose (R = 0.63, *p* < 0.01) and gender providing additional predictive information. The absence of divergence between training and validation losses over 300 epochs confirmed that the model was well calibrated and did not overfit. The MLP algorithm surrogate provides occupational health practitioners with a computationally efficient, feature-integrated tool that reproduces established radiobiological ERR/Gy projections across the full spectrum of NMHP exposure scenarios, requiring only five routinely available parameters. Application within occupational surveillance programs could support real-time, individualized radiation-induced thyroid cancer risk stratification and guide targeted protective interventions for highest-risk personnel, complementing established population-level dosimetric monitoring frameworks rather than replacing them. This model should be understood as a computational surrogate for established ERR/Gy.RST tools rather than an independent biological predictor; full clinical translation requires prospective validation against longitudinal occupational cohort data. Future iterations incorporating disaggregated internal dosimetry, genomic/post-genomic/epigenomic susceptibility markers, role-specific exposure profiles, and SHAP-based interpretability analyses will extend the model’s scope, enhance regulatory utility, and maximize its contribution to protecting the healthcare professionals who deliver metabolic radionuclide therapy.

## Figures and Tables

**Table 1:** Overview of variables.

Variable	Description
Input features	Gender of NMHP
	Distance from the gamma radiation source (in meters)
	Age at exposure
	Current age
	Cumulative absorbed doses to the thyroid (in Gy, over 5 years)
Output feature	ERR/Gy.RST

**Table 2:** Algorithm architectures and training parameters.

Algorithm	Architecture	Training Detail
Dtcr	Default parameters: single decision tree Single decision tree	Data split: 70% train, 15% test, 15% validation
Rfr	Default parameters; 300-tree ensemble	Data split: 70% train, 15% test, 15% validation estimators = 300
MLP	Input shape = 5 with Dense (64, relu) 6 hidden layers: Dense (128, relu) Dense (256, relu) Dense (64, relu) Dense (128, relu) Dense (256, relu) Dense (1, linear)	Data split: 70% train, 15% test, 15% validation epochs = 300 Optimizer: Adam Loss: MSE

*Note: ReLU = rectified linear unit.*

**Table 3:** Performance metrics of all three algorithms on the held-out test set.

Algorithm	R^2^ Score	MSE	MAE
MLP	0.999	0.002	0.010
Rfr	0.700	0.410	0.295
Dtcr	0.510	0.654	0.289

## Data Availability

Publicly downloadable data were obtained from the Radiation Effects Research Foundation (RERF) website (https://www.rerf.or.jp/ (accessed on 11 June 2026)) and used in compliance with the requirements outlined therein. More specifically, the precise dataset was accessed from https://www.rerf.or.jp/en/library/data-en/lssinc07/ (accessed on 11 June 2026). No restricted or individual-level data were accessed or utilized. The datasets were treated as open academic resources and appropriately cited. This report makes use of data obtained from the RERF, Hiroshima, and Nagasaki, Japan. RERF is a private, non-profit foundation funded by the Japanese Ministry of Health, Labor, and Welfare and the U.S. Department of Energy, the latter through the National Academy of Sciences. The conclusions in this report are those of the authors and do not necessarily reflect the scientific judgment of RERF or its funding agencies.
